# Meta-Analysis of Postoperative Adjuvant Hepatic Artery Infusion Chemotherapy Versus Surgical Resection Alone for Hepatocellular Carcinoma

**DOI:** 10.3389/fonc.2021.720079

**Published:** 2021-12-22

**Authors:** Qiao Ke, Lei Wang, Weimin Wu, Xinhui Huang, Ling Li, Jingfeng Liu, Wuhua Guo

**Affiliations:** ^1^ Department of Hepatopancreatobiliary Surgery, Mengchao Hepatobiliary Hospital of Fujian Medical University, Fuzhou, China; ^2^ Department of Hepatopancreatobiliary Surgery, Fujian Cancer Hospital, Fujian Medical University Cancer Hospital, Fuzhou, China; ^3^ Department of Radiation Oncology, Fujian Cancer Hospital, Fujian Medical University Cancer Hospital, Fuzhou, China; ^4^ Department of Interventional Radiology, Mengchao Hepatobiliary Hospital of Fujian Medical University, Fuzhou, China; ^5^ The United Innovation of Mengchao Hepatobiliary Technology Key Laboratory of Fujian Province, Mengchao Hepatobiliary Hospital of Fujian Medical University, Fuzhou, China

**Keywords:** hepatocellular carcinoma, hepatic artery infusion chemotherapy, surgical resection, overall survival, disease-free survival, meta-analysis

## Abstract

**Background:**

To systematically identify the long-term efficacy of postoperative adjuvant hepatic artery infusion chemotherapy (HAIC) for patients with hepatocellular carcinoma (HCC).

**Methods:**

PubMed, MedLine, Embase, the Cochrane Library, and Web of Science were searched to collect the eligible studies up to March 31, 2021, that compared the surgical resection (SR) versus SR+HAIC for HCC patients. The endpoints were overall survival (OS) rates and disease-free survival (DFS) rates, and the effect size was determined by hazard ratio (HR) with 95% CI.

**Results:**

A total of 12 studies (two randomized controlled trials (RCTs) and 10 non-RCTs) including 1,333 patients were eligible for this meta-analysis. The pooled results showed that OS and DFS rates in the SR+HAIC group were both better than those in the SR alone group (HR = 0.56, 95% CI = 0.41–0.77, *p* < 0.001; HR = 0.66, 95% CI = 0.55–0.78, *p* < 0.001, respectively). Furthermore, the subgroup analysis showed that patients would benefit from SR+HAIC regardless of chemotherapy regimens and courses (all *p* < 0.05), and patients with microvascular or macrovascular invasion would also benefit more from SR+HAIC in terms of OS and DFS (all *p* < 0.05).

**Conclusion:**

Postoperative adjuvant HAIC could improve the long-term prognosis of HCC patients, especially for those with microvascular or macrovascular invasion, regardless of chemotherapy regimens and courses, but it deserves further validation.

## Introduction

Hepatocellular carcinoma (HCC) is still one of the most common kinds of solid tumors, with approximately 906,000 patients being newly diagnosed to have HCC (1). Surgical resection (SR) remains the most cost-efficient curative strategy for HCC, although 50%–70% of patients have lost the chances of surgery at diagnosis (2, 3). With the development of surgical techniques and advances in perioperative management, great progress has been acquired in the prognosis of patients receiving SR. However, since the 5-year recurrence rate following SR is beyond 70% (3, 4), the long-term prognosis of HCC patients remains discouraging. Therefore, strategies intended to decrease the postoperative recurrence rate are badly warranted in clinical practice.

Numerous kinds of treatments following SR have been tried to prevent or reduce the recurrence rates, including transarterial chemoembolization (TACE), antiviral therapy, Huaier granule, interferon-α, cytokine-induced killers, and sorafenib (2, 5). But the anti-recurrence efficacy of most of the strategies has not been recognized universally, except for antiviral therapy (4). Hepatic artery infusion therapy (HAIT) followed by surgery has been confirmed in a meta-analysis to improve the overall survival (OS) and disease-free survival (DFS) of patients not candidates for transplantation (6). Hepatic artery infusion chemotherapy (HAIC), as a modality of HAIT, is first reported in 1962, but it has been flourishing in the recent decade due to the intensive chemotherapy regimen, such as FP (fluorouracil and cisplatin) and FOLFOX (fluorouracil, leucovorin, and oxaliplatin) (7). Studies have shown that HAIC is superior to sorafenib alone in the treatment of tumors resistant to multiple TACE treatments (8), combined with portal vein tumor thrombus (9) and extrahepatic metastasis (10). In addition, HAIC has also been tried in the neoadjuvant treatment with inspiring initial results (11). However, it remains controversial whether adjuvant HAIC could improve the prognosis after SR or not.

Nonami et al. (12) first identified the role of adjuvant HAIC in 1991 in a report of 19 HCC patients after hepatectomy, but the results of subsequent studies did not exactly correspond to those of a previous study. In the recent two randomized controlled trials (RCTs) (13, 14), adjuvant HAIC was found to bring survival benefits to HCC patients in both OS and DFS, but both their sample sizes are too small. Hence, we wanted to systematically review the literatures on postoperative adjuvant HAIC for HCC, and then we conducted a meta-analysis comparing the long-term efficacy of SR+HAIC versus SR alone.

## Material and Method

The systematic review and meta-analysis was registered at http://www.crd.york.ac.uk/PROSPERO/(review registry: CRD42021252416), and it was in accordance with the Preferred Reporting Items for Systematic Reviews and Meta-Analyses (PRISMA) guideline.

### Literature Search

A comprehensive literature search was conducted from January 1, 1990, to March 31, 2021, in PubMed, MedLine, Embase, the Cochrane Library, and Web of Science to identify the eligible studies, with the language confined to English only. The search strategy and MeSH terms were as follows: (“hepatocellular carcinoma” or “liver cancer” or “HCC”) AND (“hepatectomy” or “liver resection” or “hepatic resection” or “surgical resection” or “resection”) AND (“hepatic artery infusion chemotherapy” or “HAIC” or “chemotherapy” AND “prophylactic” or “adjuvant” or “postoperative”). And manual search was also conducted *via* the references of the included studies and relevant reviews to identify other potentially eligible studies.

### Eligibility Criteria

Inclusion criteria were as follows: i) patients with pathological diagnosis of HCC, ii) tumors were resectable, and iii) groups must include the SR+HAIC group and SR group.

Exclusion criteria were as follows: i) patients with other primary liver cancers or recurrent HCC, ii) patients receiving other adjuvant treatments such as TACE, iii) did not provide the data of long-term outcomes, iv) duplicate data derived from the same center, v) the articles were not written in English, and vi) abstracts, reviews, comments, letters, and case report.

### Data Extraction

According to the predefined forms, information of each study including the surname of the first author, year of publication, study design and period, and clinicopathological characteristics including sample size, tumor diameter, tumor number, microvascular invasion, macrovascular invasion, resection margin status, and chemotherapy regimens were extracted directly by two independent researchers (QK and LW). The hazard ratios (HRs) of OS or DFS were extracted from multivariate analysis or calculated by the Kaplan–Meier curves based on Engauge Digitizer 4.1 (15, 16). OS was defined as the time from resection to death or last follow-up, while DFS was defined as the time from resection to recurrence.

In case of discrepancies, an internal discussion was conducted among all the researchers, and a consensus was then reached.

### Quality Assessment

The quality of RCT was assessed according to the Cochrane Handbook (17), and a study with a total score of 0–2 was considered to be of low quality, whereas >2 was of low quality. The quality of non-randomized studies was assessed by the modified Newcastle–Ottawa Scale (NOS) (18), and a study with 0–3 stars was regarded as a low-quality one, 3–6 as a medium one, and ≥7 as a high-quality one.

### Statistical Analysis

The endpoints in this meta-analysis were OS and DFS, and effect sizes were determined by HR with 95% CI. The heterogeneity test was evaluated by χ^2^ test and *I* ^2^ statistics, and there were apparent heterogeneities if *p* < 0.10 or *I* ^2^ > 50%. When there was significant heterogeneity, the random-effects model was used to estimate the effect size; if not, the fixed-effects model was used (19, 20). Begg’s and Egger’s tests were conducted to evaluate the publication bias. Sensitivity analysis and the “trim and fill” method were performed to assess the stability of the results in this study. Besides, all the statistical analyses in this meta-analysis were performed using RevMan Version 5.3 and Stata 14.

## Results

**Figure 1:**
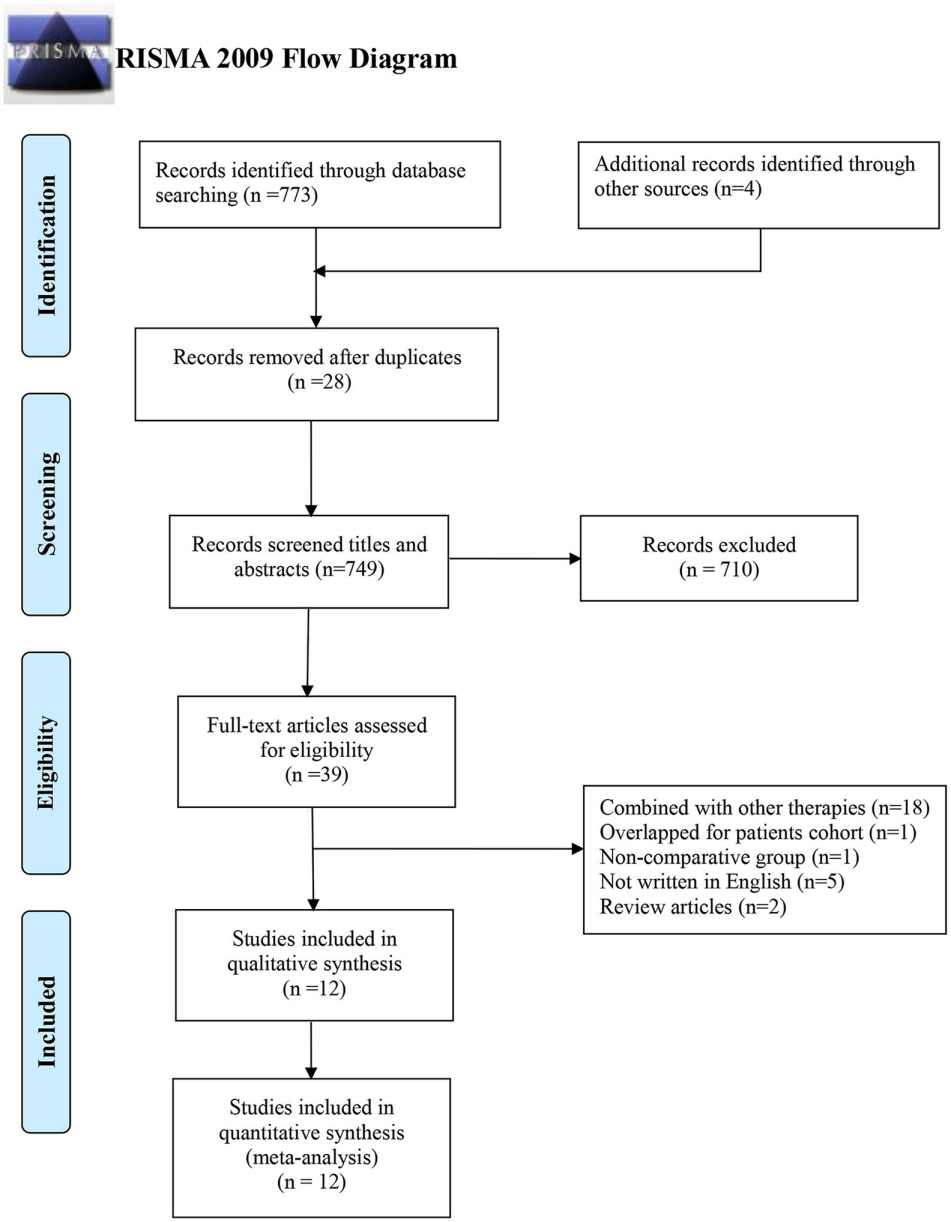
PRISMA flow diagram of studies selection. PRISMA, Preferred Reporting Items for Systematic Reviews and Meta-Analyses.

A total of 773 records were initially identified using an electronic database, as well as four more records through manual search. After 28 duplicated records were excluded, 710 records were excluded by screening titles and abstracts. Another 27 more records were excluded after reading the full text, with the following reasons: 1) 18 records of patients undergoing other combined therapies; 2) five records not written in English; 3) two records of review articles; 4) one record of patients with overlapped cohort; and 5) one record of a non-comparative group. Finally, 12 records were assessed to be eligible for this meta-analysis (12, 13, 21–30), including two RCTs (13, 26) and 10 non-RCTs (12, 21–25, 27–30) (**Figure 1**).

There were 1,333 patients enrolled in this meta-analysis, containing 466 (35%) cases in the SR+HAIC group and 867 (65%) cases in the SR group. All of the included studies were from East Asia, and 75% (8/12) came from Japan (12, 21, 22, 24, 25, 28–30). The publication year ranged from 1991 to 2020, and the earliest study start year was 1979. The sample size of each study ranged from 12 to 400. The publication information of each study and clinicopathological characteristics in each group are displayed in **Table 1**. Of note, there were apparent differences among the included studies in the median OS and DFS, which are shown in **Table 1**. The quality of each enrolled study is also exhibited in **Table 1**, among which eight were assessed as high-quality ones (13, 23–28, 30) and four as medium-quality ones (12, 21, 22, 29).

**Table 1:** Clinicopathological characteristics of the included studies.

Study	Country	Design	Study years	Treatment	Patients	Tumor size	Tumor number	Microvascular invasion	Macrovascular invasion	Mean OS	*p*-Value	Mean DFS	*p*-Value	Resection margin	Quality
						(cm)	(S/M)	(yes/no)	(yes/no)	(months)		(months)		(negative/positive)	
Nonami et al., (12)	Japan	RCS	1979–1989	SR+HAIC	19	NA	NA	NA	NA	NA	<0.001	NA	NA	19/0	M
				SR	113	NA	NA	NA	NA	NA		NA		113/0	
Niguma et al., (21)	Japan	PCS	1989–2002	SR+HAIC	6	NA	NA	NA	6/0	58.0	<0.010	15.0	<0.010	6/0	M
				SR	6	NA	NA	NA	6/0	8.0		4.0		6/0	
Tanaka et al., (22)	Japan	RCS	1998–2001	SR+HAIC	7	NA	NA	NA	7/0	NA		NA	0.940	7/0	M
				SR	8	NA	NA	NA	8/0	NA		NA		8/0	
Kim et al., (23)	Korea	PCS	2006–2008	SR+HAIC	31	4.8 ± 2.3	29/2	25/6	13/18	NA	NA	0.03	0.324	31/0	H
				SR	62	4.2 ± 2.4	60/2	43/19	14/48	NA		7.5		62/0	
Nitta et al., (24)	Japan	RCS	1997–2011	SR+HAIC	38	6.6 ± 3.9	25/13	0/38	38/0	NA	0.318	NA	0.029	33/5	H
				SR	35	7.0 ± 4.2	23/12	0/35	35/0	NA		NA		32/3	
Kojima et al., (25)	Japan	RCS	2001–2010	SR+HAIC	27	7.0 (2.8–18.0)	10/17	0/27	27/0	12.4	0.043	33.2	0.044	19/8	H
				SR	25	5.0 (1.4–17.0)	8/17	0/25	25/0	6.2		21.5		19/6	
Huang et al., (26)	China	RCT	2005–2010	SR+HAIC	42	6.2 ± 1.5	24/18	NA	NA	NA	0.028	NA	0.018	42/0	H
				SR	43	5.7 ± 1.3	23/20	NA	NA	NA		NA		43/0	
Hsiao et al., (27)	China	RCS	2006–2014	SR+HAIC	61	20/41 (≤5/>5 cm)	28/33	NA	NA	56.4	0.760	50.6	0.905	61/0	H
				SR	160	81/79 (≤5/>5 cm)	89/71	NA	NA	56.9		54.5		160/0	
Hatano et al., (28)	Japan	RCS	2001–2010	SR+HAIC	134	6.9 (1.0–25.0)	54/79	NA	134/0	28.1	0.002	9.3	0.015	113/21	H
				SR	266	7.0 (0.6–27.0)	102/164	NA	266/0	18.7		5.4		198/64	
Kuramoto et al., (29)	Japan	RCS	1997–2012	SR+HAIC	6	3.95 ± 1.57	2/4	NA	6/0	120	0.180	7.9	0.550	6/0	M
				SR	6	4.43 ± 1.80	2/4	NA	6/0	11.7		2.5		6/0	
Li et al., (13)	China	RCT	2016–2019	SR+HAIC	58	5.8 ± 0.4	36/22	58/0	0/58	NA	0.037	NA	0.023	58/0	H
				SR	58	5.5 (1.8–16.0)	42/16	58/0	0/58	NA		NA		58/0	
Hamada et al., (30)	Japan	RCS	2004–2014	SR+HAIC	37	5.6 ± 3.7	NA	37/0	37/0	NA	0.079	NA	0.172	NA	H
				SR	85	5.4 ± 3.6	NA	62/23	52/33	NA		NA		NA	

S, single; M, multiple; OS, overall survival time; DFS, disease-free survival time; RCS, retrospective cohort study; PCS, prospective cohort study; RCT, randomized controlled trial; SR, surgical resection; HAIC, hepatic artery infusion chemotherapy; NA, not available; M, medium; H, high.

The chemotherapy agents of HAIC went through three stages in the last 50 years: epirubicin-based chemotherapy regimens, cisplatin-based chemotherapy regimens, and oxaliplatin-based chemotherapy regimens. The dosages and courses of each regimen in each study are depicted in **Table 2**.

**Table 2:** The regimens and administration of HAIC in the included studies.

Study	Drugs and dosage of HAIC	Course(s)
Nonami et al., (12)	Doxorubicin (0.4 mg/kg) + mitomycin C (0.12 mg/kg) + 5-fluorouracil (250 mg/day)	5-Fluorouracil was injected 14 days, and doxorubicin and mitomycin C were injected on day 1 and day 8. 2–4 weeks after surgery, once every 3 months, a total of 4 courses.
Niguma et al., (21)	Cisplatin (5–10 mg) + 5-fluorouracil (250 mg)	Cisplatin was injected on days 1–5/7 day and continuous infusion of 5-FU for 24 h on days 1–5/7. 2–3 weeks after surgery, once every 2 or 3 weeks, a total of 4 courses.
Tanaka et al., (22)	Cisplatin (10 mg) + 5-fluorouracil (250 mg)	Cisplatin was injected on days 1–5/7 and continuous infusion of 5-FU for 5 h on days 1–5/7. 3 weeks after surgery, once every 2 or 3 weeks, a total of 4 courses.
Kim et al., (23)	Cisplatin (60 mg/m^2^) + 5-fluorouracil (750 mg/m^2^)	Cisplatin was injected for 2 h on days 2/7 and continuous infusion of 5-FU for 5 h on days 1-3/7. 4 weeks after surgery, once every 4 weeks, a total of 4 courses.
Nitta et al., (24)	Old protocol (1997–2006): cisplatin (10 mg) + 5-fluorouracil (250 mg)	Cisplatin was injected on days 1–5/7 and continuous infusion of 5-FU on days 1–5/7. 4 weeks after surgery, once every 4 weeks, a total of 3 courses.
	New protocol (2007–2011): cisplatin (60 mg/m^2^) + 5-fluorouracil (600 mg/m^2^) + mitomycin C (3 mg/m^2^)	Cisplatin dissolved in 100 ml of saline for 10 min followed by 5-FU in 100 ml saline for 10 min, mitomycin dissolved in 3 to 5 ml of saline mixed with 3 to 5 ml of degradable starch microspheres (DSMs). 4 weeks after surgery, once every 4 weeks, a total of 2 courses.
Kojima et al., (25)	Regimen 1(23 patients): course 1, cisplatin (10 mg) + 5-fluorouracil (250 mg); course 2, cisplatin (20 mg) + 5-fluorouracil (250 mg)	Course 1: cisplatin and 5-fluorouracil were injected on days 1–5, 8–12, and 15–19 for 21 days, followed by a 7-day break, followed by biweekly course 2. The target administration period was 6 months
	Regimen 2(4 patients): epirubicin (10 mg)	Once every 2 weeks, the target administration period was 6 months.
Huang et al., (26)	Oxaliplatin (85 mg/m^2^) + 5-fluorouracil (1,000 mg/m^2^) + gemcitabine (1,000 mg/m^2^)	Oxaliplatin was injected for 2 h, and 5-fluorouracil was injected for 5 h on day 1, and continuous infusion of gemcitabine over 30 min on days 1 and 8. 3 weeks after surgery, once every 4 weeks, a total of 2 courses.
Hsiao et al., (27)	Cisplatin (10 mg/m^2^) + 5-fluorouracil (150 mg/m^2^) + leucovorin (15 mg/m^2^) + epirubicin (15 mg/m^2^)	Cisplatin/leucovorin was injected for 30 min on days 1-5, 5-fluorouracil was injected for 24 h on days 1–5, and epirubicin was injected for 30 min on day 1/5. 3 months after surgery, a total of 3 courses.
Hatano et al., (28)	First course: cisplatin (10 mg) and 5-fluorouracil (250 mg) followed courses: cisplatin (20 mg) and 5-fluorouracil (250 mg)	First course: on days 1–5, 8–12, and 15–19 for 21 days, followed by a 7-day break. Followed courses: once every 2 weeks. The target administration period was 6 months.
Kuramoto et al., (29)	Old protocol (1997–2006): cisplatin (10 mg) + 5-fluorouracil (250 mg)	Cisplatin was injected on days 1–5/7 and continuous infusion of 5-FU on days 1–5/7. 4 weeks after surgery, once every 4 weeks, a total of 3 courses.
	New protocol (2007–2012): cisplatin (60 mg/m^2^) + 5-fluorouracil (600 mg/m^2^) + mitomycin C (3 mg/m^2^)	4 weeks after surgery, once every 4 weeks, a total of 2 courses.
Li et al., (13)	Oxaliplatin (85 mg/m^2^) + leucovorin (400 mg/m^2^) + fluorouracil (400 mg/m^2^) + fluorouracil (2,400 mg/m^2^)	Oxaliplatin was injected from 0 to 3 h on day 1, leucovorin was injected from 3 to 4.5 h on day 1, fluorouracil (400 mg/m^2^) was injected from 4.5 to 6.5 h on day 1, and fluorouracil (2,400 mg/m^2^) was injected over 46 h from days 1 to 2. Once every 4–5 weeks, up to a maximum of 2 courses.
Hamada et al., (30)	IA-call (65 mg/m^2^, a novel agent of high capacity dose cisplatin powder)	At least 4 weeks after surgery, only one course.

HAIC, hepatic artery infusion chemotherapy.

### Endpoints

**Figure 2:**
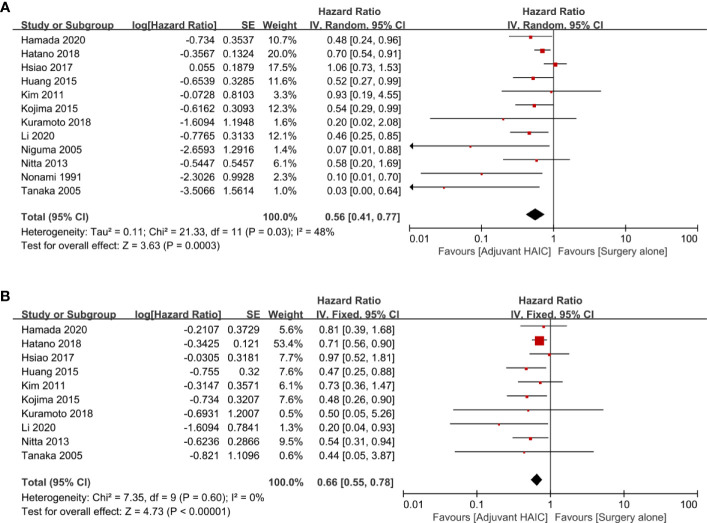
Forest plots of the overall survival and disease-free survival rates between adjuvant HAIC and surgery alone. **(A)** Overall survival. **(B)** Disease-free survival. HAIC, hepatic artery infusion chemotherapy.

**Figure 3:**
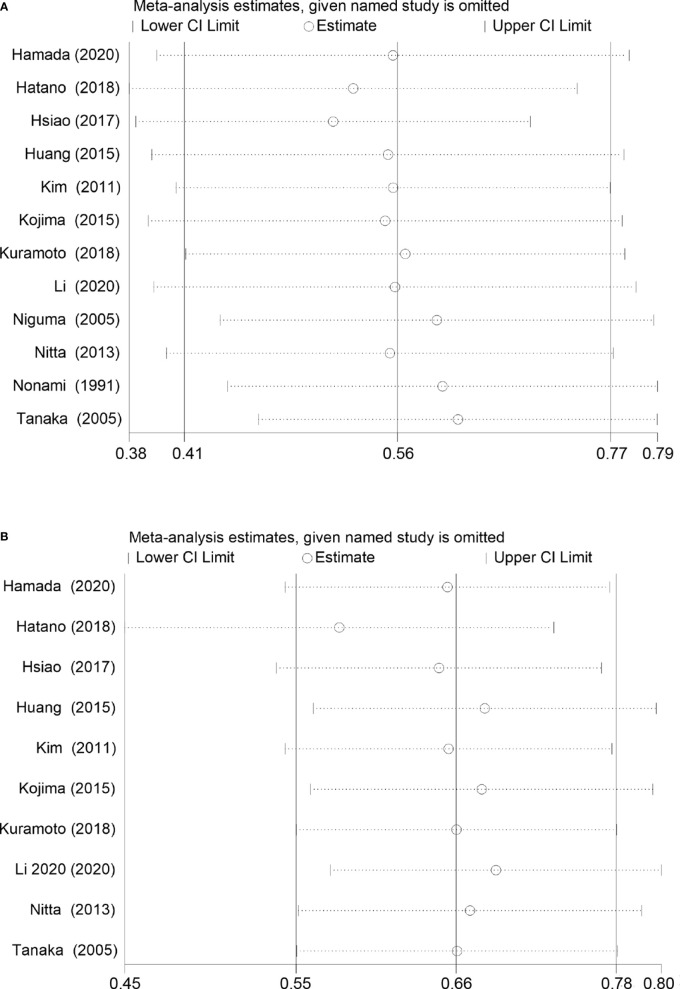
Sensitivity analysis for overall survival and disease-free survival rates in the included studies. **(A)** Overall survival. **(B)** Disease-free survival.

OS comparing SR+HAIC versus SR was evaluated in 12 included studies (12, 13, 21–30), and significant heterogeneity was displayed among the included studies (*I* ^2^ = 48%, *p* = 0.03, **Figure 2A**). The pooled HR for the median OS was significantly better in the SR+HAIC group than in the SR group (HR = 0.56, 95% CI = 0.41–0.77, *p* < 0.001, **Figure 2A**) using a random-effects model. But sensitivity analysis showed that the results did not change significantly after removing any single included study (**Figure 3A**).

DFS was compared between SR+HAIC and SR in 10 included studies (13, 22–24, 26–30). No significant heterogeneity was displayed among the included studies (*I* ^2^ = 0, *p* = 0.60, **Figure 2B**); the pooled HR for the median DFS was also in favor of the SR+HAIC group compared with the SR group (HR = 0.66, 95% CI = 0.55–0.78, *p* < 0.001, **Figure 2B**), using a fixed-effects model. And the significant difference was also confirmed in a further sensitivity analysis (**Figure 3B**).

### Subgroup Analysis of Endpoints

**Figure 4:**
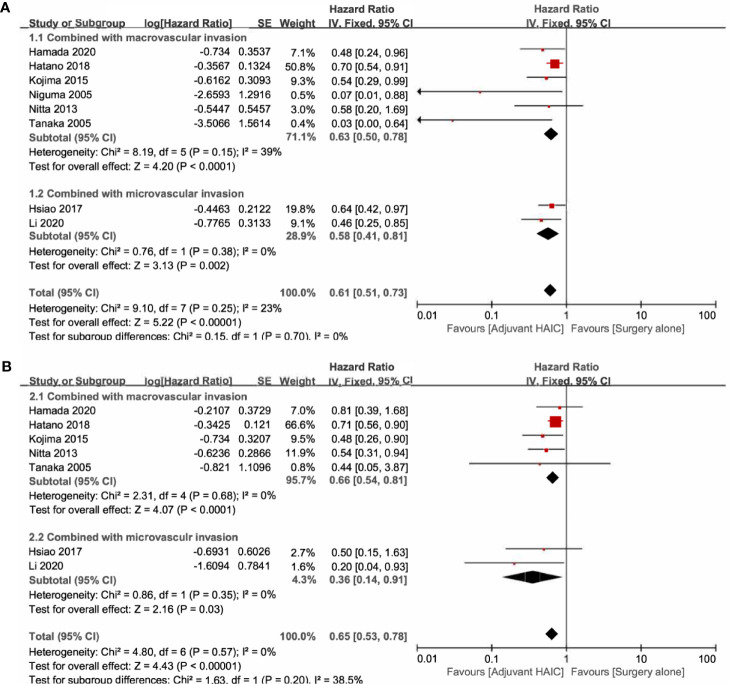
Forest plots of the overall survival and disease-free survival rates between adjuvant HAIC and surgery alone stratified by different types of vascular invasion. **(A)** Overall survival. **(B)** Disease-free survival. HAIC, hepatic artery infusion chemotherapy.

Six included studies reported the OS of patients with macrovascular invasion in the SR+HAIC group compared with the SR group (21, 22, 24, 25, 28, 30). Since no significant heterogeneity was observed (*I* ^2^ = 39%, *p* = 0.15, **Figure 4A**), a fixed-effects model was used to evaluate the pooled result. The pooled HR demonstrated that median OS was in favor of the SR+HAIC group compared with the SR group (HR = 0.63, 95% CI = 0.50–0.78, *p* < 0.001, **Figure 4A**). DFS of patients with macrovascular invasion was compared between SR+HAIC and SR in five included studies (22, 24, 25, 28, 30), and a similar advantage was also observed (HR = 0.66, 95% CI = 0.54–0.81, *p* < 0.001, **Figure 4B**).

There were two studies focusing on the subgroup of patients with microvascular invasion (13, 27). Significant heterogeneity was not observed between the two included studies (*I* ^2^ = 0, *p* = 0.38, **Figure 4A**), and the pooled HR for the median OS was in favor of the SR+HAIC group compared with the SR group (HR = 0.61, 95% CI = 0.51–0.73, *p* < 0.001, **Figure 4A**) using a fixed-effects model. A similar finding was observed in the pooled HR for the median DFS (HR = 0.36, 95% CI = 0.14–0.91, *p* = 0.03, **Figure 4B**).

A subgroup analysis was also conducted, which was stratified by the study design (prospective vs. retrospective), sample size (<100 vs. ≥100), chemotherapy regimen (cisplatin-based vs. oxaliplatin-based), and course (≤2 vs. >2). The results showed that the advantage of SR+ HAIC over SR alone was also observed in terms of both the median OS and DFS in all the subgroup analyses (all *p* < 0.05, **Table 3**).

**Table 3:** Subgroups analysis stratified by different factors.

Subgroups	Overall survival				Disease-free survival			
	Studies included	Effect model	HR (95% CI)	*p*	Studies included	Effect model	HR (95% CI)	*p*
Microvascular invasion	2	Fixed	0.58 (0.41–0.81)	0.002	2	Fixed	0.36 (0.14–0.91)	0.030
Macrovascular invasion	6	Fixed	0.63 (0.50–0.78)	<0.001	5	Fixed	0.66 (0.54–0.81)	<0.001
Prospective study	4	Fixed	0.48 (0.32–0.74)	<0.001	3	Fixed	0.52 (0.33–0.82)	0.005
Retrospective study	8	Random	0.59 (0.40–0.87)	0.007	7	Fixed	0.69 (0.57–0.83)	<0.001
Sample < 100	7	Fixed	0.49 (0.34–0.72)	<0.001	6	Fixed	0.53 (0.39–0.73)	<0.001
Sample ≥ 100	5	Random	0.63 (0.42–0.94)	0.020	4	Fixed	0.73 (0.59–0.90)	0.003
Cisplatin based	9	Random	0.63 (0.44–0.89)	0.009	8	Fixed	0.69 (0.57–0.83)	<0.001
Oxaliplatin based	2	Fixed	0.49 (0.31–0.76)	0.002	2	Fixed	0.42 (0.23–0.74)	0.002
Courses ≤ 2	3	Fixed	0.49 (0.33–0.71)	<0.001	3	Fixed	0.54 (0.34–0.85)	0.008
Courses > 2	9	Random	0.58 (0.38–0.89)	0.010	7	Fixed	0.68 (0.56–0.82)	<0.001

HR, hazard ratio.

### Complications

Most of the complications were mild, such as transient fever, tolerable nausea and vomiting, loss of appetite, and mild aspartate aminotransferase/alanine aminotransferase (AST/ALT) elevation. No lethal complications were reported in all the included studies, but Nitta et al. (24) reported that five patients (13%) experienced grade 3/4 complications, and Kojima et al. (25) observed a persistent grade 3 myelosuppression. The details of complications are described in **Table 4**.

**Table 4:** The complications of adjuvant hepatic artery infusion chemotherapy.

Studies	Complications
Nonami et al., (12)	No serious complications were observed. Some patients complained of transient fever or uncomfortable feelings.
Niguma et al., (21)	No serious complications were observed. The most common adverse reactions were tolerable nausea and loss of appetite.
Tanaka et al., (22)	No serious complications were observed.
Kim et al., (23)	No serious complications were observed.
Nitta et al., (24)	No lethal complications were observed, but five patients (13%) experienced grade 3/4 adverse events.
Kojima et al., (25)	Two patients were observed to have related complications: one developed grade 2 acute kidney injury and one had persistent grade 3 myelosuppression.
Huang et al., (26)	No serious complications were observed. The most common adverse reactions were tolerable nausea and/or vomiting.
Hsiao et al., (27)	No serious complications were observed. The common adverse reactions were nausea, vomiting, and mild AST/ALT elevation.
Hatano et al., (28)	Not provided.
Kuramoto et al., (29)	No serious complications were observed.
Li et al., (13)	No serious complications were observed. The common adverse reactions were pain, vomiting, hypoalbuminemia, thrombocytopenia, anorexia, leukocytopenia, and hyperbilirubinemia.
Hamada et al., (30)	Not provided.

AST/ALT, aspartate aminotransferase/alanine aminotransferase.

### Publication Bias

**Figure 5:**
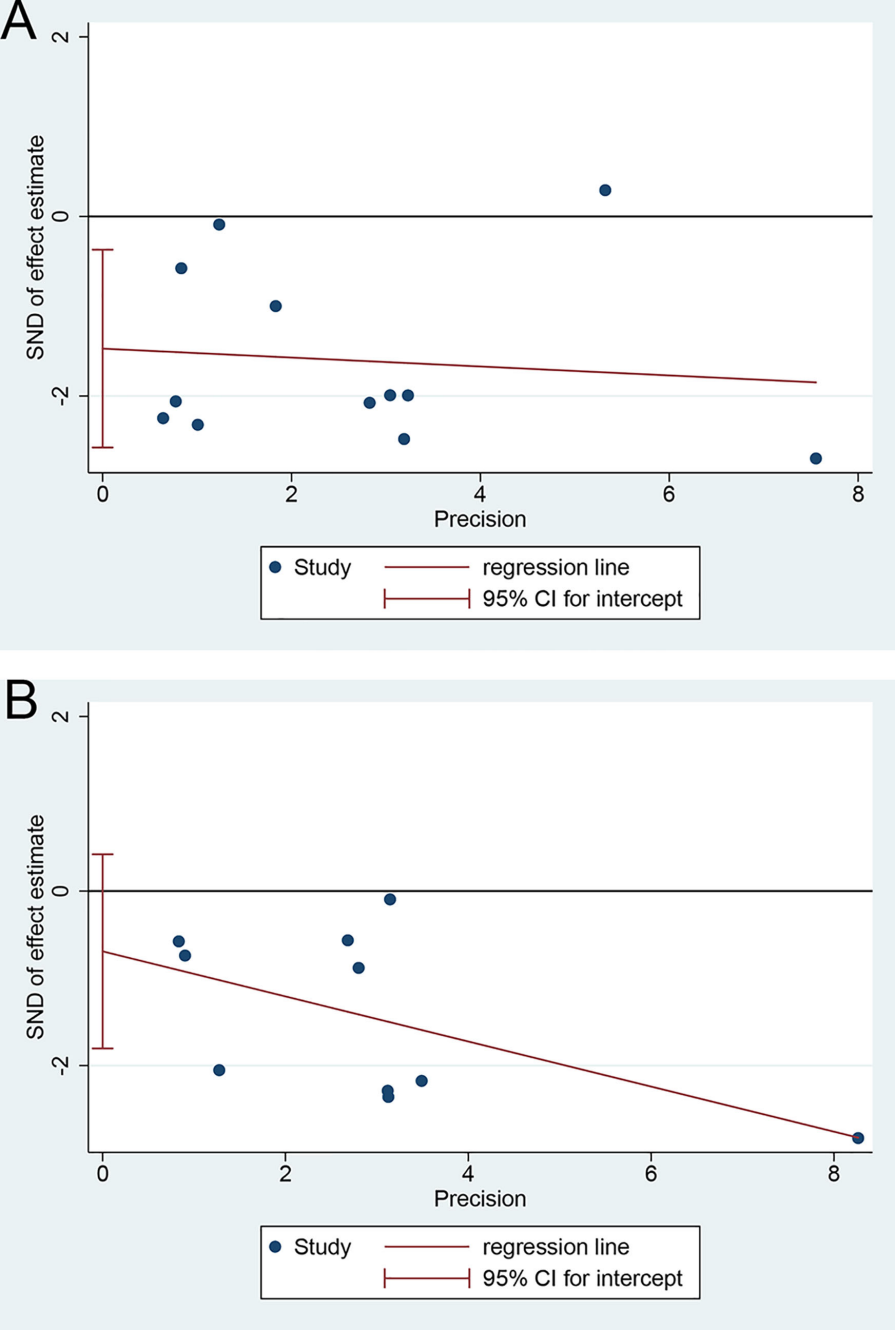
Egger’s test for publication bias. **(A)** Overall survival. **(B)** Disease-free survival.

There was an apparent publication bias in the pooled HR for the median OS using Egger’s test (*p* = 0.014, **Figure 5A**) but not Begg’s test (*p* = 0.054). But the advantage of SR+HAIC over SR alone remained (HR = 0.577, 95% CI = 0.427–0.780, *p* < 0.05) after using the “trim and fill” analysis, which suggested that the unpublished studies might have few effects on the results. On the other hand, there was no significant publication bias noted in the pooled HR for the median DFS, using Egger’s test (*p* = 0.190, **Figure 5B**) and Begg’s test (*p* = 0.592).

## Discussion

This is the first meta-analysis aiming to evaluate the long-term efficacy of postoperative adjuvant HAIC for resectable HCC patients. A total of 12 studies with 1,333 patients were identified to be eligible for this article, and results showed that adjuvant HAIC could improve both OS and DFS of patients receiving SR compared with SR alone. Furthermore, the advantage of adjuvant HAIC was also confirmed using the subgroup analysis stratified by the risk factors such as microvascular or macrovascular invasion, study design, sample size, chemotherapy regimen, and course.

Recurrence is still the “Achilles heel” of the postoperative management for HCC (31, 32). TACE is preferred in East Asia to prevent recurrence after SR, especially in China (33, 34). But there are several disadvantages in adjuvant TACE. On the one hand, the anti-recurrence efficacy of adjuvant TACE remains controversial, especially in Europe and the United States (4); on the other hand, TACE was reported to induce recurrence *via* upregulation of hypoxia-inducible factor-1a and vascular endothelial growth factor related to embolization (35, 36). HAIC might be an alternative to TACE in the following aspects: 1) HAIC could significantly increase the total dose of chemotherapy and prolong the exposure time of high-concentration chemotherapy drugs, and 2) HAIC could prevent adverse events related to embolization such as embolization syndrome and ectopic embolism. Some studies have shown that HAIC is more effective than TACE in the treatment for unresectable advanced HCC with a higher objective response rate (37, 38). In this study, the advantage of adjuvant HAIC over SR alone has been confirmed in both OS and DFS, but it still lacks a direct comparison of adjuvant HAIC versus adjuvant TACE.

As is known to all, one size does not fit all. Adjuvant TACE has been recommended by Chinese guidelines on the postoperative management of HCC for patients with high-risk factors, such as tumor diameter >5 cm, macrovascular invasion, microvascular invasion, and incomplete capsule (2), although it still lacks strong evidence. Likewise, adjuvant HAIC could not benefit all HCC patients receiving SR. In this meta-analysis, we found that patients with microvascular or macrovascular invasion would benefit more from adjuvant HAIC, and the reasons might be as follows: 1) hematogenous spread and metastasis are more likely to occur in patients with vascular invasion, and 2) compared with conventional TACE, continuous HAIC can maintain higher local concentrations of chemotherapeutic drugs and eliminate potential micrometastasis, resulting in fewer recurrence or metastasis and prolonged survival time. However, other potential candidates should be explored in the future.

Chemotherapy regimens played a decisive role in the efficacy of HAIC (39). Earlier failure of HAIC might be due to the single drug infusion, such as epirubicin. An intensive regimen of two or three chemotherapy agents has shed light on the renewed interest in HAIC, such as FP and FOLFOX. In this study, both cisplatin-based and oxaliplatin-based regimens were identified to be efficient in the improvement of long-term prognosis, but the optimal regimen remains unknown. Of note, increased chemotherapy means more risk of toxicity, and there is a ceiling effect to some drugs. Fortunately, in phase I and phase II clinical trials of of HAIC combined other treatments such as IFN-α, sorafenib, lenvatinib, apatinib, sintilimab, and toripalimab, encouraging results in the recent years were found (40–42), and we expected more results from the ongoing trials.

Catheterization of HAIC has always been a concern among surgeons and physicians. The preferred catheterization technique is like TACE, and HAIC is re-inserted into the appropriate position and extubated after drug injection. This repeated intubation is complicated and expensive, but it could guarantee a precise catheter position each time (40). Another catheterization technique is described as follows: the gastroduodenal artery and the right gastric artery are embolized, and then the catheter is connected to the intrahepatic artery to inject drugs *via* the subcutaneous infusion port. This technique is more feasible and costs less, but the catheter position could not be adjusted in time, and the incidence of serious catheter-related complications is as high as 12% (43). However, there are no studies comparing directly the two different catheterization techniques.

Several limitations should be noted in the current study. First, 75% (8/12) of the included studies were retrospective, which hints that selection and recall bias were hard to avoid. Second, the Child–Pugh grade, alpha-fetoprotein (AFP) level, tumor size, and tumor number were reported to be associated with the response rate of HAIC, but we have not performed a corresponding subgroup analysis due to relevant missing data. Third, the chemotherapy regimens and courses were a little different from those of included studies, although we conducted a subgroup analysis stratified by the above factors. Fourth, data on salvage treatment after recurrence were not available, which might influence the long-term survival. Finally, all the enrolled studies came from East Asia, which indicates that the results may not be applicable for patients from Western countries.

## Conclusion

With the current data, we conclude that postoperative adjuvant HAIC could improve the long-term prognosis of HCC patients, especially for those with microvascular or macrovascular invasion, regardless of chemotherapy regimens and courses, but it deserves further validation. In the future, the improvement of catheterization technique, optimization of chemotherapy regimens, screening of potential beneficiaries, and combination with other treatments are the exploration directions of adjuvant HAIC.

## Data Availability Statement

All data included in this study are available upon request by contact with the corresponding author WHG (email: mailto:guowuhua@aliyun.com).

## Author Contributions

QK, LW, and WMW: acquisition of data, analysis, and interpretation of data. XHH and LL: conception and design of the study. QK and LW: drafting of the article. JFL and WHG: critical revision and final approval. All authors contributed to the article and approved the submitted version.

## Funding

This work was supported by the social development medical project of Fuzhou, Fujian, PRC (2018-S-103-5), the Joint Funds for the Innovation of Science and Technology of Fujian Province, Fujian, PRC (2017Y9117), and the Key Clinical Specialty Discipline Construction Program of Fuzhou, Fujian, PRC (201912002).

## Conflict of Interest

The authors declare that the research was conducted in the absence of any commercial or financial relationships that could be construed as a potential conflict of interest.

## Publisher’s Note

All claims expressed in this article are solely those of the authors and do not necessarily represent those of their affiliated organizations, or those of the publisher, the editors and the reviewers. Any product that may be evaluated in this article, or claim that may be made by its manufacturer, is not guaranteed or endorsed by the publisher.
